# Implementation challenges of electronic blood transfusion safety systems: Lessons from an international, multi‐site comparative case study

**DOI:** 10.1111/tme.13095

**Published:** 2024-09-09

**Authors:** Stijn Horck, Nick Fahy, Trisha Greenhalgh

**Affiliations:** ^1^ Department of Health Promotion, Faculty of Health, Medicine and Life Sciences Maastricht University Maastricht The Netherlands; ^2^ RAND Europe Cambridge UK; ^3^ Nuffield Department of Primary Care Health Sciences University of Oxford Oxford UK

**Keywords:** bedside‐scanning, implementation, organisational learning

## Abstract

**Background:**

Severe transfusion reactions resulting from errors in matching the correct blood with the correct patient are considered never events. Despite the relative technical simplicity of barcode scanning for patient‐blood bag matching, the adoption and universal application of this safety measure are by no means universal. This study highlights the logistical and institutional challenges associated with spreading, scaling up, and sustaining such IT‐supported safety measures in healthcare.

**Study Design and Methods:**

We report findings from a 5‐year, prospective, multi‐site case study conducted across one hospital in England and three hospitals in the Netherlands. Ethnographic methods, including interviews and observations, were used at each site to investigate the implementation of barcode scanning‐supported safety pathways for blood transfusions.

**Results:**

Significant variation was observed across the sites in the adoption and implementation of barcode scanning‐supported safety pathways. Despite the potential for reducing transfusion errors, the introduction of this innovation was met with varying levels of success in different settings.

**Discussion:**

This study highlights the critical role of inter‐hospital learning and flexible system design in successfully implementing barcode scanning‐supported safety pathways for blood transfusions. A more structured, national‐level network for knowledge sharing could enhance the spread and sustainability of such innovations across healthcare settings.

## 1 INTRODUCTION

In recent years, there has been a global increase in the integration of Information Technologies (IT) to improve blood transfusion services within hospitals. This growth has been accelerated by the active endorsement and support from supranational organisations such as the World Health Organization^1^ and national entities like Serious Hazards of Transfusion,^2^ recognising the significant benefits that such technologies contribute to the safety and efficiency of the blood transfusion process. This particularly holds for the bedside and lab processing scanning innovations. Innovative hospitals have led the development of bedside scanning systems, pioneering the adoption of technologies that enable comprehensive tracking, often referred to as ‘vein‐to‐vein’ systems.^3^ These systems are rooted in the combination of existing IT frameworks and the tangible infrastructure within hospitals, which has inevitably resulted in various systems that, while aiming to fulfil the same function, differ significantly in their structure, development and implementation processes. For clarity and ease of understanding, we frame these varied bedside scanning innovations under the umbrella term ‘electronic blood transfusion safety systems’ (EBTSS).

Scientific articles describing the development and implementation processes of EBTSS predominantly focus on one hospital without considering the influence of, or learning from, hospitals that have previously adopted these systems.^4^, ^5^, ^6^ Consequently, if there is a lack of both scientific studies and evaluations comparing the development and implementation processes of EBTSS across hospitals, there is a chance of missing shared learning opportunities among hospitals, even internationally. Therefore, this innovation serves as an ideal subject for a case study in implementation research with a specific focus on health care innovation. It is particularly fitting to using analysis frameworks to examine the various domains and subdomains where EBTSS can offer insights for learning across different health care organisations. This paper uses the Consolidated Framework for Implementation Research (CFIR) by Damschroder et al.^7^ for a comprehensive analysis of the challenges encountered during the implementation phases of EBTSS and determining which challenges could be universally addressed by adopting solutions that have been successful in other hospitals.

The contribution of the study is three‐fold: first, to identify universal challenges in the implementation of EBTSS and to describe solutions that are not constrained by the specific type of EBTSS in use. Second, to highlight possible strategies for knowledge dissemination between hospitals regarding these systems. Third, to investigate the (international) differences in EBTSS among hospitals. Therefore, the results of this study will provide health care managers with more concrete and practical guidance for EBTSS implementation and integrate these insights with pre‐existing implementation guidelines such as those described by Jones et al.^3^ As we compare in this study hospitals in the Netherlands with a hospital in the UK, the results also touch upon the international knowledge exchange between hospitals.

## 2 MATERIALS AND METHODS

### 2.1 Electronic blood transfusion management systems

Bedside scanning innovations for blood transfusions involve an IT‐based system where health care workers use handheld devices to electronically verify the match between a patient, the blood product and the nurse at the point of care. Upon scanning the patient's wristband and the blood product's barcode, the system checks for compatibility and records the transfusion details automatically in IT systems (electronic patient records [EPR] and/or lab IT‐systems). Therefore, the processes of blood ordering, delivery requests and performing a blood transfusion follow digital checks if these processes are integrated within these systems. While some hospitals have digitised nearly all these processes, there are variations. For example, in the case of blood delivery requests, some hospitals require their staff to order digitally but then physically collect the blood from the lab. Others use a pneumatic mail system (‘tube mail’), and some have smart fridges on the wards that include digital checks as well. An EBTSS may range from a comprehensive closed‐loop system to a system with a more modest IT integration aimed at enhancing safety and efficiency in the blood transfusion process. These (comprehensive) system designs lead to an increase in patient safety by preventing transfusion errors, streamline the workflow by reducing manual checks and paperwork, and ensure compliance with regulatory standards. For further understanding of EBTSS, we refer to.^3^, ^4^, ^6^, ^8^, ^9^ In this study, only hospitals with an EBTSS that incorporates bedside scanning were included. Bedside scanning requires a robust IT infrastructure, and its successful implementation signifies a considerable degree of IT integration and a change in workflow for health care workers involved in blood transfusions.*

### 2.2 Origin and governance of the study

The study originated as a MSc dissertation project and extended to a PhD, focussing on learning reactions and behaviours within and between health care organisations, funded by ZonMw. The UK‐based empirical work was conducted as an attachment to the Oxford Biomedical Research Centre (BRC) as part of a wider theme focused on innovation which was led by TG and overseen by an independent external advisory group. The Netherlands‐based work followed the BRC's protocol design, but was tailored specifically for the Dutch setting and this particular study, making it distinct and not part of any broader research themes or projects in the Netherlands.

Ethical approvals for this study were two‐fold. The UK work was covered by the University of Oxford's Central University Research Ethics Committee.^10^ The Dutch research received approval from the Ethical Review Committee Inner City faculties (ERCIC) of Maastricht University.

### 2.3 Study design

This study adopted an international multi‐case methodology to conduct a detailed comparative analysis of the implementation process of EBTSS across three hospitals. Complementary (less detailed) data were gathered from one additional hospital, a blood bank and a technology vendor to enhance the richness of the findings.

Multiple qualitative data collection methods were employed; in‐depth semi‐structured interviews, group interviews, non‐participant observations and document analysis. The interviews were conducted with various groups of participants involved with the EBTSS of their respective hospitals, covering roles such as project team members, haemovigilance officers, clinical chemists, laboratory specialists, analysts, transfusion technicians, nursing staff and nurse leaders, physicians (haematologists), IT and functional application managers, and support staff from technology vendors. The selection of these respondents was purposeful done by the contact person of the hospital—a clinical chemist, a project team member or a haemovigilance officer. The interview questions were designed around themes derived from implementation literature^7^, ^11^ and were conducted in either English or Dutch. The non‐participating observation visits were made to day‐care units, emergency rooms and blood laboratories, lasting from one to four and a half hours. Detailed field notes were taken, meticulously recording direct observations and informal interactions with staff members. Most data was collected by Stijn Horck, an implementation scientist with social science rather than clinical expertise. As a result, Stijn Horck focused more on the specifics of working with an EBTSS than on clinical details of the blood transfusion process. This means that the reported benefits and drawbacks of working with an EBTSS are based solely on respondent descriptions and objective observations, without the researchers' personal reflections. Scientific articles, reports by the hospitals, trusts and department of Health and briefings between management describing the implementation process and results of the system have been analysed. These were either selected by the research team or provided by some of the respondents. Table 1 provides an overview of the data sources, the settings from which data was collected, the nature of the data, and the types of EBTSS present in this study.

**TABLE 1:** Overview of data collection.

Data source	Data collected	EBTSS*
Hospital A		
Academic hospital (English)	(Group) interviews (14): Project management team members, haematologists, nurses, nurse leaders, IT managers, Nurse Information Officer, in‐house vendor employees, registrar.	Category 3
Collected: March–May 2019	Observations (2): Day care unit (3.5 h) Training (1.5 h)	Since: 2013
Hospital B		
Academic hospital (Dutch)	Interviews (10): Project team members, initiator, IT‐manager, clinical chemists, nurse specialist, nurses, haematologist, lab manager, functional application manager, haemovigilance officer	Category 3
Collected: November–December 2022	Observations (2): Day care unit (4 h) Emergency department (1 h)	Since: 2017
Hospital C		
Peripheral hospital (Dutch)	(Group) interviews (10): Project team members, clinical chemist, nurses, nurse leaders, lab specialist, lab analyst, haematologist, haemovigilance officer, functional application manager	Category 2
Collected: February 2023	Observations (2): Day care unit (3.5 h) Blood transfusion lab (1.5 h)	Since: 2022
Hospital D		
Peripheral hospital (Dutch)	Interviews (2): Haemovigilance officer, clinical chemist**	Category 1
Collected: March 2023	Observations (1): Blood transfusion lab together with day care unit (4.5 h)	Since: 2022
Technology vendor		
EPR supplier (Dutch)	Group interview (1): EPR developer, Lab IT integration specialist	‐
Collected: April 2023		
Blood bank		
National Blood Bank (Dutch)	Interview (1): Hospital relation manager	‐
Collected: April 2023		

*Note*: The time disparities in data collection can largely be attributed to the COVID‐19 pandemic.

Abbreviation: EBTSS, electronic blood transfusion safety systems; IT, Information Technologies.

* The year since the eventual category of EBTSS was implemented, not the year the hospital started bedside scanning.

** At the time a representative of the Dutch Association for Blood Transfusion.

### 2.4 The different categories of EBTSS

To contextualise the EBTSS in the studied hospitals, a categorisation of these systems has been made based on the analysis of three primary hospitals. Additionally, a smaller case study of a fourth hospital provided further insights, to validate to some extent whether our categorisation and understanding of the EBTSS implementation processes were applicable beyond the three primary hospitals. This approach resulted in the following categorisation, which considers both the eventual form as well as the intermediate developmental stages of the EBTSS.

#### 2.4.1 Category 0

This category represents the absence of an EBTSS, serving as the baseline from which all hospitals begin the digitalization of their blood transfusion management. In this stage, the process of blood administration is entirely paper‐based between the lab IT systems and the EPR.

#### 2.4.2 Category 1

This category involves using the scanning program, which is part of the lab IT system, at the patient's bedside for scanning purposes. This setup demands minimal IT integration but significantly alters the workflows of health care workers. Often, nurses must adapt to using a different IT system for scanning purposes. This approach is typically a preliminary step due to the existing scanning capabilities within lab IT systems for inventory management, minimising the need for extensive IT modifications.

#### 2.4.3 Category 2

Similar to category 1, this EBTSS version involves embedding the scanning function within the EPR. EPR technology vendors, who design modules within the EPR to generate orders and process lab IT system data for scanning purposes, usually drive this integration. In our data, it appears that for hospitals implementing this EBTSS type, it often represents the final stage of their EBTSS development.

#### 2.4.4 Category 3

**FIGURE 1:**
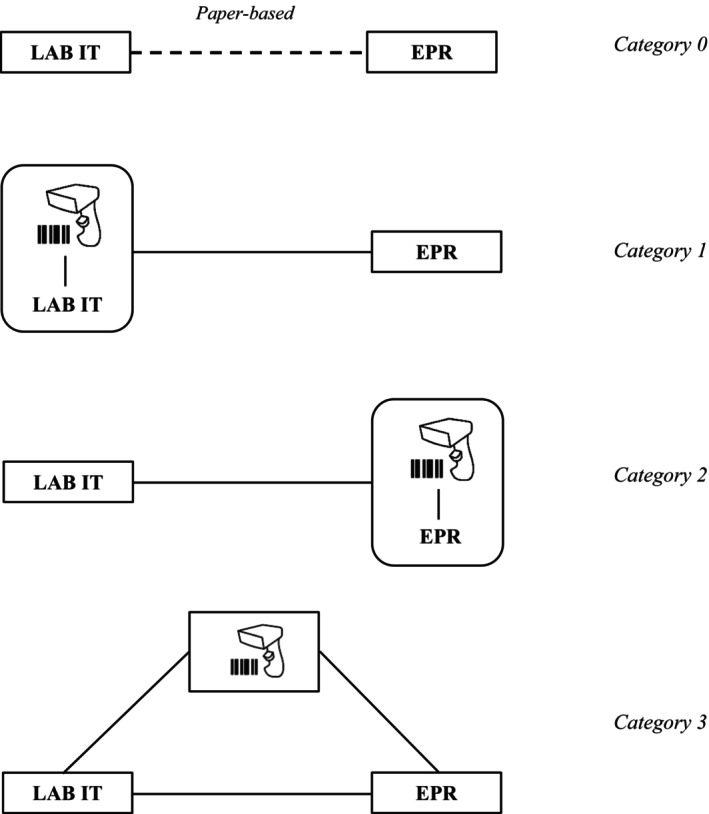
Simplified overview of the different electronic blood transfusion safety systems categories. This is a simplified overview of the various approaches to Information Technologies (IT) integration and does not cover all the processes involved in blood transfusions. A more detailed description would require inclusion of steps such as blood ordering, bedside activities and the procedures for issuing or remotely collecting blood. EPR, electronic patient records.

This is the most IT‐intensive category, featuring separate, often custom‐designed software integrations between the scanning system, EPR and laboratory IT systems. The advantage of this complex technical approach is the reduced reliance on the typically standard content provided by EPR and lab IT system technology vendors. It enables processes without necessitating health care workers to familiarise themselves with entirely new IT systems. However, the possibility of developing and implementing such an EBTSS depends on the presence of enough financial support and in‐house IT expertise. Furthermore, it is characterised by a significantly longer implementation period. A schematic overview of the categories is shown in Figure 1.

### 2.5 Data analysis

The data analysis was conducted in three stages after all interviews were transcribed verbatim, except for two interviews from hospital D, which were part of the observation phase and thus only consisted of field notes. The first stage of analysis involved an open coding process, focusing on identifying elements that described the specifics of each hospital's EBTSS, followed by a thematic analysis to differentiate EBTSS types based on technical features and developmental progress (resulting in Figure 1). In the second stage, a new round of coding was performed by applying the CFIR by Damschroder et al.^7^ CFIR guided the identification of sub‐domains that offer amendable approaches in the implementation process of EBTSS. This approach enabled a thorough comparison between the hospitals on the five domains of the CFIR: (1) innovation: the implemented EBTSS; (2) outer setting: the broader context surrounding the hospitals; (3) inner setting: the specific environment of a hospital where the EBTSS is put into practice; (4) individuals: the roles and characteristics of the people involved; and (5) implementation: encompassing the various activities and strategies used to implement the EBTSS. This resulted in a detailed examination of the implementation similarities and differences across various domains and subdomains for each hospital. The third stage entailed investigating the highlighted dimensions for each hospital and pinpointing the decisions made to overcome implementation challenges. These decisions were then further analysed to determine whether they were open choices or constrained by path dependency. This analysis was crucial in understanding the range of options available to hospitals in addressing specific challenges.

## 3 RESULTS

This section is divided into two parts. First, for each dimension of the CFIR, we present our findings by detailing the specific aspects of that dimension concerning the EBTSS, followed by a discussion of the overarching challenges encountered during the implementation of EBTSS, along with the strategies hospitals have employed to address these challenges. Notably, the focus lies on whether these solutions are constrained to the unique context of each hospital or if they are more openly applicable. Second, we look into the influence of the network and stakeholders in facilitating the spread of knowledge regarding these challenges and solutions among the hospitals as well as the extent of international sharing of this knowledge.

### 3.1 The CFIR domains

#### 3.1.1 The innovation domain

EBTSS, even in their less comprehensive forms, constitute complex innovations. While the technology itself is relatively straightforward, aligning its use with existing work processes and practices presents significant challenges. In the innovation domain, we focus on the particular ways in which these practices are integrated with IT system implementations. Hospitals A and B invested significant efforts into integrating various IT systems within their respective IT infrastructure, while hospital C faced challenges in making even relatively simple adjustments within their IT‐systems. Notably, each hospital developed its EBTSS independently, with limited external reference points. Although hospital B attempted to replicate another hospital's EBTSSS, the specific shape and form of the system changed during implementation. It had to be reinvented to fit the specific organisational and social structure of hospital B. Therefore, the implementation of an EBTSS is not a simple ‘plug‐and‐play’ adaptation, but a socio‐technical innovation.^12^ This suggests a prevailing need for tailor‐made flexibility in EBTSS implementation, while also highlighting the challenge of not knowing exactly what will work in different hospital settings.

There are distinct differences among the hospitals regarding the innovation dimension. For example, the role of smart fridges is crucial in hospital A but less so in hospital B and even absent in hospital C. These different approaches could be explained by the latter hospitals' reliance on extensive pneumatic tube systems, which negates the need for smart fridges in their case. In contrast, hospital A views smart fridges as a critical element in transforming blood transfusion practices, as it forces the health care workers to work differently. The design of the innovations also varies, particularly regarding handheld devices and the information displayed during blood transfusions. Hospital A uses advanced scanner handhelds that show comprehensive information, whereas hospitals B and C require a separate computer during transfusions. Differences also emerge in the relative advantages of EBTSS implementation. Although enhancing patient safety is a common goal across all hospitals, hospital A places considerable emphasis on cost‐saving by reducing blood usage, a factor highlighted in their (scientific) report publications. In contrast, hospitals B and C, possibly due to the lower frequency of blood transfusions in the Netherlands, do not focus on this aspect. Finally, a comparison of category 3 EBTSS reveals that the vendor choice has an impact on the adaptability and customization of EBTSS solutions. Both hospitals A and B cite successful customizations due to contracting smaller, more flexible technology vendors. In contrast, hospital C's experience with a major EPR technology vendor was marked by rigidity, with respondents attributing this to the vendor's focus on standard content.

##### Challenges and solutions

In the implementation of EBTSS, all hospitals face challenges in two areas of the innovation domain: (1) the integration with pre‐existing IT systems and (2) limitations of operational components. The ability of hospitals to address these challenges varies, often constrained by the specific features and characteristics of their EBTSS and existing infrastructure. Integration with existing IT systems is an unavoidable hurdle, regardless of the EBTSS type pursued. Some IT‐related respondents mentioned that certain systems are more fit for integration than others. However, given that the blood transfusion process is a relatively minor component of EPRs and lab IT‐systems, completely replacing these systems for optimal EBTSS integration is often impractical. Consequently, hospitals generally have to work with the systems they already possess. Hospitals A and B, for instance, responded to the inflexibility by self‐designing systems to enhance interconnectivity and collaborating with smaller technology vendors to assist them.

There weren't many companies around to choose from, but when I talked to this particular company, (…) they were willing to start again. They weren't in just selling marketing mode; they recognised that what they had was not going to be good enough, and so they were willing to work with us and it worked out really, really well—Haematologist and project lead Hospital A.

Our lab IT supplier is not very big; it currently serves the blood transfusion services for two others, I believe. Yes, it's much smaller in scale, and we are a very important partner in that. So, if we want to have something changed, it goes much more easily than it would through a major IT supplier*—*Clinical chemist Hospital B.

Hospital C, on the other hand, was limited by the capabilities of its current EPR vendor and lacked the resources for finding self‐designed solutions. A common hardware issue across all hospitals was the compatibility of wristbands and scanners. The UK hospital A led a national initiative for new wristband designs, a challenge which was not as prominent for the Dutch hospitals because wristbands had already been updated in the Netherlands before they started to develop their EBTSS. Nonetheless, all hospitals had to enforce stricter wristband protocols, as wristbands are essential for patient identification. These protocol developments, while specific to each hospital, offer transferable solutions. However, in practise there was hardly any transfer of these solutions to other hospitals. The choice of scanners varied, with each hospital eventually converging on more universal models compatible with both wristbands and blood bag barcodes when replacements were necessary. For example, they chose scanners that could also read newer technologies like QR codes, which were introduced in the early 2000s and are now used in various hospital processes. This approach ensured consistent scanning capabilities across different hardware.

#### 3.1.2 The outer setting domain

In the outer setting domain, the most significant differences emerge, largely influenced by the type of hospital and the national health care system in which it operates. Despite early external pressures, such as the EU directive in the early 2000s focusing on blood transfusion safety enforcing the presence of haemovigilance officers (Directive 2002/98/EC), hospitals reported low societal or political pressure to develop and implement a digital system assisting the blood transfusion administration. However, hospitals had to incorporate various national and international guidelines, regulations and professional recommendations into their EBTSS, such as transfusion protocols based on haemoglobin values.

A critical aspect within this domain is the relationship each hospital has with its technology vendors, which varies considerably. Hospital A maintains a unique, service‐based partnership with its vendor, an arrangement with in‐house vendor employees. Hospital B, on the other hand, has multiple vendors through its IT department, coordinating between EPR and lab IT system vendors to manage their custom IT‐integrations. Hospital C initially had a close collaboration with its EPR vendor during the development of its blood transfusion module. Overtime, however, this relationship has since changed to a more traditional customer relation where the hospital is just a customer and is largely responsible for determining the most effective way to use and improve their EBTSS within their specific context.

In the beginning, during the project, it was weekly; now it is every 6 to 8 weeks. We still have meetings with them about some improvement points we have (…) I think from the signals we receive that they are just happy with what it currently is—Haemoviligance officer Hospital C.

We have placed this specific commercial dimension within this domain of CFIR, although we have reservations about whether this or the other domains truly capture the interaction between a hospital and a commercial vendor in this regard. Interestingly, all hospitals report that interactions with other hospitals regarding EBTSS are primarily facilitated by their vendors through ‘user groups’, where they tend to provide knowledge to other hospitals rather than taking over the lessons learned from others, except for hospital B, which has made substantial changes based on the insights from another academic hospital in the Netherlands. A notable international difference lies in the funding of EBTSS innovations. For the UK hospital A, financial support is heavily reliant on boards, committees and other authoritative entities within their trust, whereas the Dutch hospitals B and C financed their EBTSS internally, based on their own investment strategies. This difference in funding mechanisms might partly account for the shorter implementation process in the Dutch hospitals compared with hospital A.

##### Challenges and solutions

The integration within IT systems, as mentioned in the previous domain, is also the main challenge in the external domain. However, in this domain, the challenge particularly refers to persuading technology vendors to accommodate specific requests for modifications of the hospital. Each hospital has developed distinct strategies to address these requests, consisting of both path‐dependent decisions as well as some unconstrained approaches. Hospital A enforced immediate support from their technology vendor through their service‐based contract. Aiming to establish itself as a global leader in EBTSS, the hospital actively engaged various stakeholders, building a supportive network within influential committees of the trust. As an academic institution, it also spread its achievements through trust reports and scientific publications, thereby becoming a flagship for its technology vendor and gaining leverage for enhanced collaboration. In contrast, the Dutch hospitals B and C had to approach their relationships with technology vendors differently, as these vendors generally preferred offering standard content, instead of personalised systems. According to the respondents from the latter hospitals, changes within the system required broad support from other users. Both hospitals leveraged their contacts, largely facilitated by the vendors, to form support coalitions, eventually achieving the critical mass needed for the vendors to modify their software.

And in those user groups, if there is something that affects multiple hospitals, or if there is something that hasn't been done yet, or if there is a question from a hospital, we address it in the user group, like, ‘You know, that would be something nice’. It is then developed into something like, well, a concept or a prototype or whatever—Nurse Hospital C.

Particularly in the Netherlands, two vendors, along with a few smaller ones, dominate the market for EPR software and other IT support systems. In contrast, the market for hardware‐based technologies, including handheld scanners, is more varied yet appears equally accessible to both Dutch and UK hospitals. An interesting aspect of this challenge is that standard content within these systems often must be adapted to changing national guidelines. This occurred twice in both hospitals B and C, which used the integration of updated national guidelines to strengthen their arguments for a required adjustment to the vendors, thereby inducing necessary changes in the standard content.

#### 3.1.3 The inner setting domain

It is evident that there are notable similarities in certain aspects of the hospitals' internal settings. These similarities are particularly prominent in communication processes, both within the health care workforce and in the formal and informal relationships, networks and department specific teams that span the internal environment of each hospital.

A defining characteristic of EBTSS is its ongoing development, with the involved health care workers continuously aiming for improvements. All hospitals in this study have implemented comprehensive feedback mechanisms to gather suggestions for improvement, which are communicated by the departments or teams as demands for further action of IT and technology vendors. The effectiveness and consistency of this communication are vital across all hospitals, despite variations in their specific feedback structures due to differences in physical, IT and work infrastructure. However, the nature of communication directed towards the health care workforce is not the same for every hospital. The mission alignment and relative priorities regarding the implementation of EBTSS appear to differ significantly. In hospital A, the necessity to develop strong business cases for implementation, expansion and scaling processes is evident, which has most likely contributed to a clear understanding of the mission among project management, IT departments and clinical staff. The three primary benefits mentioned for EBTSS implementation are enhanced patient safety through improved tracking, increased efficiency by replacing the four‐eyes‐principle with a single nurse and a computer and cost savings due to reduced blood use. Yet, in the Dutch hospitals B and C, nurses and physicians were often uncertain about the primary objectives of the higher management in implementing EBTSS. While they could assume that safety and efficiency improvements were likely goals, the exact priorities were unclear. Interestingly, as mentioned in the interviews as well as seen in the observations, the four‐eyes principle remains prevalent in these hospitals. This persists even though reliance on the EBTSS for verification is allowed under both their internal protocols and the national guidelines.

Just because of the business, you naturally see it happen too. What you then naturally think is, well, that's a reliable colleague, so it should all be fine. And that four‐eyes principle almost becomes a three‐eyes principle, like: yes, I bring my colleague along, that's fine, and go ahead—Nurse Hospital B.

With the package cell, you go to the room together with another colleague, and there you scan the product. You search in the EPR, and you click on ‘ready to receive’. Then you enter that system and scan it (…). You get a pop‐up, and there they need to enter yours or you colleague's name and login code, and so on.—Nurse leader Hospital C.

##### Challenges and solutions

A common problem in the inner setting across all hospitals was the training of staff in using the EBTSS. Initially, all three hospitals adopted a ‘train‐the‐trainer’ strategy, but this quickly proved to be ineffective. The large academic hospitals A and B, with access to more resources, outsourced part of the training to external parties. In contrast, hospital C, not being a large academic institution, lacked this option and faced ongoing challenges in adequately training its nursing staff, particularly those who perform blood transfusions on rare occasions. Another issue was the misalignment of expectations among nursing staff in the Dutch hospitals due to unclear communication about the objectives of EBTSS, leading to varying compliance rates. While hospital B had made progress in rectifying this, hospital C was still addressing the initial negative perceptions, worsened by the initial difficulty in using the EBTSS and unclear objectives of procuring and implementing EBTSS. A key lesson here is that poor goal‐setting communication and limited knowledge accessibility regarding EBTSS can significantly delay achieving high compliance rates and beneficial outcomes. Strategies to mitigate this delay included facilitating communication between wards and the blood lab for queries and establishing key users on the wards to provide immediate support to colleagues. Furthermore, managing relational disruptions appeared to be a critical factor. The introduction of EBTSS directly affected relationships within the health care workforce. For instance, the shift to digital blood ordering altered the dynamics between clinicians and lab personnel. Initially, there was confusion and frustration due to incomplete blood orders, as scheduling information required by the EBTSS was not readily available to physicians but was completed by secretaries.

The ordering process doesn't exactly follow the procedure we use in the hospital. For example, when I request a blood transfusion, I actually have to immediately fill in the day, date, and time when the transfusion should be given. And, of course, I don't know that (…) I now go to the secretary, who then handles everything else—Haematologist Hospital C.

Well, that physician says: ‘YES, but I made the request!’ But we don't see it, we don't see it. Because, well, it's not completed, you know. But: ‘YES, I did fill it in, and it's always such a hassle and…’ Well, then we could look in orders in the EPR and we would see that it wasn't fully completed. So, just to get rid of the nagging, you would take it over. Well, that's not allowed, of course, because it hasn't been officially finalized—Lab analyst Hospital B.

Often, if there's a problem with a transfusion or, um, then they often call the lab first saying: I can't manage it, or something like that. Then we can give tips, because there are cheat sheets written for all the departments. So we say: yes, if you first open that and then do what it says [laughter]. It usually works then—Lab analyst Hospital C.

Over time, as awareness of the new process grew, these frustrations disappeared. This underscores the importance of addressing changes in professional relationships and the work routines they influence when implementing EBTSS.

#### 3.1.4 The individual domain

The implementation of EBTSS in hospitals necessitated a change in skill demands and roles within the health care workforce. The individual domain reveals considerable similarities across the studied hospitals, especially regarding the impact on specific groups and individuals.

Nurses, as the primary users, are most significantly affected, needing to incorporate bedside scanning and new IT systems into their daily practices. While laboratory personnel, physicians and IT managers also experience changes in their work, their core work routines remain largely unchanged. Across all hospitals, the nursing staff faced challenges adapting to the technology, because the design and implementation of EBTSS was often not aligning well with their capabilities or needs. For instance, nurse leaders in hospital C initially found the EBTSS burdensome and unreliable due to technical issues. Over time, proficiency with the EBTSS improved across all hospitals, with hospitals A and B now reporting 90%–95% compliance among nurses. The perception of the system's necessity also gradually evolved among all staff categories. Interestingly, no respondents expressed a desire to go back to the previous way of monitoring, managing and performing blood transfusions. Lab personnel, in particular, appeared to be highly satisfied with the system. The EBTSS has proven especially beneficial for haemovigilance officers, aiding them in obtaining comprehensive overviews for reporting safety issues in the blood transfusion chain. Within the individual domain, the involvement of leadership in the development and implementation of EBTSS did vary among the hospitals. Hospital A's successful implementation is for the greater part attributed to a renowned haematologist who initiated the EBTSS, leveraging his influence for continued funding and management support. Hospital B established a project team with clear roles and autonomy in decision‐making from higher management. In contrast, hospital C's approach involved a clinical chemist and haemovigilance officer leading the project team, who also decided on the inclusion of representatives from all key professional groups in the project team. They also relied on key users to facilitate implementation in their wards, though the perception of the effectiveness of this strategy varied among respondents from hospital C. However, hospital A positively reviews these key users, although here framed as ‘ward champions’.

##### Challenges and solutions

The implementation process can benefit from actively creating new roles tailored to individuals' capabilities and needs. The three hospitals demonstrate various strategies for doing so. For instance, hospital A appointed ‘ward champions’ out of the informal or formal leaders within a department, hospital B integrated former nurses as functional application managers to bridge the gap between nursing and IT, and hospital C identified IT‐savvy nurses to assume formal key‐user positions. Moreover, leveraging influential individuals to advocate for EBTSS can be highly effective. For instance, hospital A capitalised on the prominent status of a haematologist, while hospital B involved board members in forcing technology vendors to implement necessary adjustments within their software when the project team's efforts alone were insufficient.

So, you kind… you use like a, you know, a great doctor, like (…), the one that drives change. It's kind of like, you know, you can kind of throw the name around that like, you know, we've got this consultant onboard with this, and they lead on it—Project management team member Hospital A.

The IT provider was very protective at first. Initially, they really wanted to enter a university hospital to handle everything, that's how it was… But we had to demand certain modules be turned off via the board of directors. That wasn't easy, I don't know how they feel about it now, but at the time, I think we were somewhat unique in that—IT manager Hospital B.

A central takeaway for this domain is the apparent autonomy hospitals have in determining who is in charge of the development and implementation process, selecting opinion leaders and creating other supportive roles. This flexibility in role assignment and creation seems crucial in tailoring the EBTSS implementation process to the specific needs and contexts of individual hospitals.

#### 3.1.5 The implementation domain

In all three hospitals, the EBTSS that is currently in place deviated to some extent from the originally planned system, highlighting the crucial role of adaptability for successful implementation. Various subdomains of the implementation process, many of which were not overly constrained, influenced this adaptability.

A common feature across the hospitals was the establishment of feedback mechanisms, recognising the importance of involving all hospital staff engaged with EBTSS. The feedback was particularly vital in emergencies, where the initial EBTSS designs did not cope with the time pressure and amount of blood needed in these situations. Notably, all hospitals mentioned especially in this area having employed a ‘trial‐and‐error’ approach. The strategy for rolling out EBTSS also showed similarities, with a department‐by‐department implementation prioritising areas with the highest blood use. However, a notable oversight in the pre‐implementation assessment of needs and context was the inadequate consideration of the primary EBTSS users: the nurses. While project groups and committees within all hospitals included a broad spectrum of professionals, nurse input was typically minimal. Although this was less true for hospital A. The involvement of nurses in providing feedback for EBTSS improvements primarily occurred post‐implementation. The approach to engaging nurses varied among the hospitals. Hospital A's top‐down strategy primarily involved informing nurses rather than seeking their active input. Hospital B focused on the user‐friendliness of the system to naturally foster staff commitment but still missed direct engagement with nurses beyond those from high‐use departments. Hospital C, reflecting on its process, recognised that nurses were involved too late in the project, highlighting a significant gap in achieving nurse engagement.

##### Challenges and solutions

The main challenge faced by all hospitals in the study was to engage the nursing staff in using the EBTSS. Achieving this level of active participation was crucial for encouraging nurses to contribute to the ongoing improvement of the system. To tackle the primary issue of engaging nursing staff in the use of EBTSS, hospitals adopted various strategies. Hospital A enforced strict compliance by mandating specific training modules, with the consequence of nurses being restricted from using the smart fridge if they did not complete the training. This approach, though challenging, eventually led to high compliance among nurses.

So, once you've got a certain percentage of like, OK, we can see this is working, then there's making them mandatory and saying, ‘We're not going to accept the alternative, which is what you were doing before’. And we've done that with sample acceptance, so we don't accept samples unless they've got a label from a track—Project management team member Hospital A.

Hospital B adopted a similar tactic, refusing blood delivery requests done through the old procedure. In contrast, hospital C chose a less coercive approach, emphasising collaboration with nursing staff for joint system development. However, the level of management support for this partnership varied, as reported by the nursing staff at hospital C.

I think, well, it was fairly late when I actually heard about it, that a blood transfusion module was coming. Then I was approached by the functional administrator: hey, would you like to take a look at the transfusion module, because you will be working with it too. (…) Well, then they eventually gave a demo, and I said: Okay, this looks like this, but I see this and this and this and this, and I see this and this and this. Have you thought about that? Um, no, oh? Does that happen then? Yes, that does happen—IC Nurse Hospital C.

Then, there have been all kinds of different working groups within the hospital, where they thought: the day ward is simple, we'll do that last. Well, it turned out to be the most complicated. So yes, we have met very often and yes, we had to work out all the patient flows and paperwork and everything involved, which was quite a task—Nurse Hospital C.

An additional focus was on leveraging nurses' expertise, particularly during emergencies, to adapt and refine the EBTSS. For hospitals B and C, this involved several workgroup sessions to modify the EBTSS for efficiently handling large‐volume blood transfusions in short periods. However, the initial lack of nurse engagement seems to be caused by the inadequate involvement of nurses in the EBTSS's development and early implementation phases.

I'm now really curious if any improvement is actually being felt, so to speak, because of this. Or if they say: it was a big hassle, and it's still not working properly.

I:‘So, the system first had to undergo a sort of reputation recovery?’

‘Yes, yes, you immediately start 3‐0 behind. Yes, very much so (…) I think we are almost at 0‐0 now, so to speak. And I would really like to move to an advantage, a lead’—Clinical chemist Hospital C.

This indicates that a more effective solution for fostering nurse engagement would be to involve them more in the pre‐implementation phase, rather than relying on the post‐implementation corrective measures taken by the hospitals.

Table 2 outlines the common challenges and their respective solutions that are either minimally or not constrained by the type of EBTSS and hospital setting. This table highlights areas where learning opportunities are most prevalent as the ‘sweet spot’ for knowledge sharing and application.

**TABLE 2:** Overview of common challenges and solutions to address them.

CFIR domain	Challenges	Solutions
Innovation	IT‐systems integration	Tailoring EBTSS features to specific needs (i.e., the necessity of smart fridges)
	Compatibility of hardware	Following national wristband guidelines and adopting universal scanners (that aligns with economic replacements)
		Consider wristband protocols in other hospitals
	Vendor rigidity	Seeking smaller, more adaptable technology vendors
Outer setting	Vendor rigidity	Engage in contracts suitable to the hospital (i.e., service‐based, product procurement)
		Leveraging user groups and networks to enforce software changes
		Use national guidelines as a pressure tool
	Funding	Utilising different financial strategies
Inner setting	Goal misalignment among primary users	Clear communication about the necessity of implementing EBTSS
	Ineffective training approaches	Considering multiple training options (train‐the‐trainer for less technical based, outsource training for complicated work routine changes)
	Work relations disruptions	Increase awareness of changed processes among the entire health care workforce
Individual setting	Deciding leadership	Leverage the influential statuses of involved individuals
		Construct the project group to consist of all groups impacted by EBTSS
	Bridging nursing and IT	Integrating former nurses as functional application managers
	Creating supportive staff	Appoint ward champions from individuals in (in)formal positions per ward/department
		Create key users based on IT‐savvy nurses to give hands‐on assistance in the wards/departments
Implementation	Engaging nursing staff in using EBTSS	Strict compliance enforcement through training requirements (i.e., locking smart fridges)
		Refusing old procedures to be processed
		Emphasise the nursing staff's role in continuous development
		Involve the nursing staff in the pre‐implementation phase
	Adapt to emergencies	Ensure consistent management support (i.e., post‐implementation workgroups)
		Utilise the safe and efficient workarounds driven by expert knowledge of nurses

Abbreviations: CFIR, Consolidated Framework for Implementation Research; EBTSS, electronic blood transfusion safety systems; IT, Information Technologies.

### 3.2 The role of external parties in sharing lessons learned nationally and internationally

Here we focus on knowledge information streams between hospitals included in this study. During the data collection phases, it became clear that there are three key stakeholders significantly involved with EBTSS on the national level: the national blood banks, the technology vendor and the professional association for blood transfusion. These parties hold divergent views on their roles in disseminating lessons learned from implementing EBTSS. First, we will briefly explore the roles and perspectives of these three stakeholders. However, due to data access limitations, particularly during the COVID‐19 pandemic, our focus is primarily on these three stakeholders in the Dutch context. Subsequently, we will examine some of the international knowledge exchanges observed among the hospitals.

The hospital relationship manager at the Dutch national blood bank acknowledged their awareness of the various systems, which helps in identifying hospitals suitable for testing innovative approaches that require detailed reporting from a well‐designed EBTSS. They recognise common problems solved elsewhere but do not view themselves as central coordinators for sharing these solutions. However, they occasionally direct hospitals to other hospitals facing similar issues. The technology vendor highlighted that most problems, especially those related to system adaptability, stem from hospitals using lab IT systems with incompatible coding software, complicating system integration. They encourage hospitals who want to use their bedside‐scanning modules to collectively pressure these lab IT systems to enhance their connectivity options. The vendor's role in solution sharing is confined to their user groups, without seeing a need for a more active role in fostering national EBTSS learning capacity. The clinical chemist of hosptial D, who was at the time a representative of the Dutch Association for Blood Transfusion, mentioned that the current focus of the association is on how different systems align with national guidelines. They acknowledged the yet unaddressed task of contributing to a national learning curve for EBTSS implementation, indicating an area for potential future involvement.

Both nationally and internationally, we did not find a systematic infrastructure that facilitates inter‐organisational learning for EBTSS. However, certain elements within the different implementation narratives suggest that some forms of system learning have occurred. Some members of the project team in hospital B remembered visiting hospital A in the UK well before their own EBTSS was even an idea. Interestingly, despite ending up with similar systems, the overall impression was that they could design a better system compared to what they had observed there. It appears that hospital B was primarily motivated by noticing the aspects of hospital A's EBTSS that were not effective, without being aware of how hospital A would, or eventually did, resolve these issues. Another more indirect example is the lesser problems faced by Dutch hospitals B and C regarding wristbands compared with hospital A. Hospital A struggled with non‐standardised wristbands, complicating their EBTSS implementation and development. Hospitals B and C, who developed their EBTSS later, benefited from more uniform wristband developments. This progress can be seen as an example of system learning, which does not refer to a transfer of knowledge from hospital to hospital. The regulatory change extends beyond inter‐organisational learning. It involves learning at the level of the entire system, which includes policies, laws, markets and stakeholders that together form the framework of how health care is organised in a country and the international relations outside of the country. Beyond these learning examples, a broader international learning development can be recognised. Over time, there has been a noticeable improvement in hardware technologies used in the EBTSS processes (i.e., scanners, computers, handhelds). These advancements have made, for example, scanners more adaptable to various requirements and simplified their usage for nurses. Since these scanners are primarily used in different industries, this type of system learning extends beyond the EBTSS learning network and the health care sector, falling into a wider, global inter‐industry learning movement.

Finally, as mentioned earlier, a common trend was observed across all three hospitals. They view their role more as disseminators of knowledge rather than as collectors of knowledge from others. This tendency may partly stem from our sample, which likely includes hospitals more inclined to share their experiences. However, considering their participation in knowledge‐sharing groups, it raises a question about whether this attitude of prioritising knowledge provision over seeking new insights is prevalent among hospitals in the context of EBTSS and potentially other similar innovations in health care settings.

## 4 CONCLUSION AND DISCUSSION

The widespread adoption of EBTSSs in various countries marks a significant advancement in health care. EBTSS not only plays a crucial role in enhancing the safety and efficiency of blood transfusion chains but also serves as an empirical example of inter‐organisational learning and ‘system learning’ within health care, a concept not extensively explored. This study highlights the importance of inter‐hospital learning, as some implementation challenges of EBTSS took considerable time to resolve, often with solutions already existing elsewhere. The insights into common challenges and flexible solutions could be beneficial even before EBTSS implementation, aiding hospitals in evaluating their readiness for EBTSS adoption. For instance, not addressing specific issues pre‐implementation might lead to suboptimal outcomes, as noted by Hibbs et al.,^4^ who identified the lack of impact on blood transfusion practices due to insufficient training, support and feedback. The comparison of EBTSS implementation across different hospitals reveals that common challenges exist, necessitating flexible system design tailored to each hospital's context. Such flexibility often requires self‐design and collaboration with technology vendors for necessary adjustments. Critically, the continuous inclusion of primary users, such as nursing staff, in the design process is essential to ensure the EBTSS meets their needs and capabilities. The CFIR is a valuable analysis framework that has helped categorise and organise the specific elements explaining why the development and implementation of the EBTSS were similar in some ways and varied in others. However, we found that the CFIR is not all‐encompassing, as some elements were difficult to categorise within its domains. For instance, viewing the commercial partnerships between the vendor and hospitals as stakeholder relations within the outer setting lacks contextual depth. The NASSS framework provides a more suitable domain for this aspect of innovation implementation by incorporating the value proposition of the innovation.^11^ This approach provides better guidance for understanding how value is generated for the involved stakeholders.

One limitation of this study is that the initial development stages of the EBTSS at hospitals A and B occurred over 15 years ago. This has resulted in significant recall bias and a very small sample of available respondents, as many have retired or changed jobs. This contrasts with the rich descriptions of how the EBTSS currently operates in these hospitals, and illustrates the challenges of researching lengthy innovation processes. Another limitation is that we approached hospitals to investigate their EBTSS, even though EBTSS is a term defined by us. Initially, it took some time for respondents to fully understand what we meant by EBTSS that goes beyond just the scanning activities. This likely led to some relevant statements being omitted, but it also expanded our understanding of the EBTSS through unforeseen elements mentioned by respondents.

This research has identified specific solutions to these challenges, suggesting that greater progress could be achieved if even the unique hurdles and solutions were readily accessible for inter‐hospital learning. While system learning does occur, it is currently limited to user groups. National‐level organisations could play a role in collecting and disseminating these lessons, but currently, none see this as their responsibility. Simple inter‐hospital learning experiences, like the open discussion of the preliminary results of this study during a national consortium of blood transfusion professionals in the Netherlands, highlighting the wristband protocol issue, showed the potential for easy exchange of insights. Acknowledging the challenges each hospital might face, a better‐organised learning network could bridge these knowledge gaps. The summary of common challenges provided in this study represents a first step in consolidating learning before, during and after EBTSS implementation. However, establishing a real learning network requires the commitment of all parties involved in all facets of the electronic management of blood transfusion practices.

## AUTHOR CONTRIBUTIONS

Stijn Horck is the lead researcher of the study. He was involved in collecting data from all participants and observations, managed and stored the data, recruited the Dutch hospitals, conducted the primary data analysis, coordinated communication between the research team and respondents and wrote the main text along with the revisions. Nick Fahy supervised the UK part of the study. He participated in data collection in the UK, co‐designed the data collection and analysis strategy, contributed to interpreting the results and assisted in writing and revising the study. Trish Greenhalgh initiated the study as part of a project under the Oxford Biomedical Research Centre, which she leads. She integrated the study into the implementation literature, contributed to interpreting the findings and assisted in writing and revising the study.

## FUNDING INFORMATION

This study is part of a PhD dissertation funded by ZonMw (grant number 165720001).

## CONFLICT OF INTEREST STATEMENT

The authors have no competing interests.

## Data Availability

The data of this study are stored on the secure drives of Maastricht University, according to its Research Data Management Code of Conduct. The data can only be made available for the review purposes of this paper (contact the corresponding author Stijn Horck).

## Footnotes

* Hospitals that only use inventory lab scanning without bedside scanning were not included, as these systems do not demand substantial IT integration with EPR or significant alterations to health care workers' tasks.
